# The potential of smoke-free products to reduce harm for smokers: what does the toxicological evidence say?

**DOI:** 10.1007/s11739-025-04093-0

**Published:** 2025-08-19

**Authors:** Reinhard Niessner

**Affiliations:** Chair of Analytical Chemistry and Water Chemistry, Institute of Hydrochemistry, Technical University of Munich, Lichtenbergstr. 4, Garching, 85748 Garching, Germany

**Keywords:** Toxicology, Smoke-free nicotine product, E-cigarette, Heated tobacco product, Oral nicotine product

## Abstract

There continues to be a high prevalence of smoking in many European countries. In Germany, for example, there are over 20 million smokers, most of whom exhibit little desire to quit. In other countries, the adoption of smoke-free products (SFPs), including e-cigarettes (ECs), heated tobacco products (HTPs), and oral nicotine pouches (NPs), is helping smokers to transition away from cigarettes. In Germany, debate about SFPs primarily focuses on their potential harms to non-smokers, particularly the underage population. This debate seems one-sided: raising concerns alone does not sufficiently inform the 20 million smokers about the comparative health risks of cigarettes and SFPs, an issue increasingly echoed by practitioners and researchers. Instead, the current discourse is dominated by misconceptions, as evidenced by surveys on smokers’ perceptions of the relative health risks of cigarettes and SFPs. Considering the gravity of the topic, it is essential to revisit the scientific facts. The growing evidence shows that SFPs, including ECs, HTPs, and NPs, expose users to significantly fewer numbers and lower concentrations of toxicants relative to combustible cigarettes. In vitro studies and biomarkers of harm in SFP users suggest that these lower emissions translate to reduced risks of harm. It is the nature of science that the evidence will never be complete, but the totality of data should be considered when discussing the correct handling of SFPs. At present, these data suggest that SFPs can play a useful role in curbing the individual and societal risks associated with smoking.

## Introduction

The prevalence of smoking remains high in several European countries, including Germany, Greece and Spain, with many smokers showing little inclination to quit. In other countries, the acceptance of smoke-free products (SFPs), such as e-cigarettes (ECs), heated tobacco products (HTPs), and nicotine pouches (NPs) is gradually reducing smoking incidence. For instance, in the United Kingdom, where SFPs are promoted as a lower-risk alternative, smoking prevalence among adults (≥ 18 years) has fallen from 20.3% in 2010 to 11.9% in 2023 [1]. In Sweden, the availability of SNUS is suggested to have been a major factor in Sweden’s record-low smoking prevalence [20] and in the US the decline of smoking prevalence was linked to the uptake of e-cigarettes among adults [10].

In Germany, the debate about SFPs is skewed towards their potential harms, especially among non-smokers including underage individuals [e.g. [11]]. These concerns are valid and underscore the importance of regulatory measures to prevent youth access. Yet, it is equally important to acknowledge the scientific evidence indicating that these products present a significantly reduced risk profile relative to cigarettes. Hence, for adult smokers who are unable or unwilling to quit, SFPs offer a potentially less harmful alternative to combustible tobacco. This nuanced perspective, however, is often overshadowed by a discourse that emphasizes the risks without adequately considering the potential benefits for harm reduction.

It is important to emphasize that the primary cause of smoking-related diseases is not nicotine itself, but the toxicants present in cigarette smoke [24]. Inhalation products (ECs, HTPs) and oral products (NPs) are smoke-free, meaning that they do not burn tobacco, thereby also eliminating the sidestream smoke. This is no minor detail regarding the formation and release of harmful substances. When tobacco is ignited, various classes of reactions take place (e.g., combustion, pyrolysis, oxidation, thermal decomposition, and free radical formation); consequently, cigarettes emit ~ 6500 components [21], of which ~ 100, termed harmful and potentially harmful constituents (HPHCs), are of toxicological concern. As SFPs do not burn tobacco, they emit significantly lower levels and numbers of HPHCs than combustible cigarettes [12, 19]. This crucial point is often overlooked in discussions, while a key issue remains unaddressed: in countries like Germany, as many as one-third of adults still smoke with little desire to quit cigarettes [5], the most harmful type of nicotine delivery product.

Based on my knowledge as an aerosol chemist with extensive experience in measuring harmful substances in cigarette smoke and HTP aerosols, this rather one-sided discourse surprises me. In the early 1990 s, my Institute (Institute of Hydrochemistry at the Technical University of Munich) developed and tested a photoelectric on-line aerosol sensor to measure polycyclic aromatic hydrocarbons (PAHs) in cigarette smoke [18]. At the same time, the first articles were published about the predecessors of modern HTPs, already attesting their potential to reduce emissions relative to combustible cigarettes. Considering that notable technological innovations in SFPs have been made since then, I suspect the differences in toxicant emissions between SFPs and combustible cigarettes are significant.

To address the one-sided debate on the harms of SFPs, I have evaluated current data on the relative toxicity of SFP aerosols and extracts for three different SFPs: ECs, HTPs, and NPs. As a comprehensive analysis and comparison of all HPHCs is beyond the scope of this short article, I focus on two classes of toxicants emitted from combustible cigarettes: tobacco smoke are tobacco-specific nitrosamines (TSNAs) and polycyclic aromatic hydrocarbons (PAHs).

Four TSNAs, *N*-nitrosonornicotine (NNN), 4-(*N*-methylnitrosoamino)−1-(3-pyridyl)−1-(butanone) (NNK), *N*-nitrosoanatabine (NAT), and *N*-nitrosoanabasine (NAB), comprise one of the most important classes of carcinogenic chemical found in tobacco smoke and deserve careful consideration. They are produced during the fermentation process of tobacco, where nitrate species interact with the tobacco alkaloids nornicotine, anatabine, and anabasine. Insufficient combustion of the tobacco in cigarettes, coupled with the presence of amines and NOx, will also lead to the formation of these toxicants of primary concern.

PAHs do not occur naturally in tobacco, but can originate from the curing process if the tobacco is exposed to exhaust gases from wood or organic fuel heat sources. PAHs are also formed during the incomplete combustion of tobacco leaves during cigarette smoking. Over 500 different PAHs have been identified in the mainstream smoke of a cigarette and many are classified as carcinogens; in particular, benzo[a]pyrene (BaP) is classified as a Class-1 carcinogen [21].

In short, this Point of View seeks to evaluate recent scientific evidence on the toxicity of SFPs, specifically in comparison to combustible cigarettes. I first assess the number and levels of harmful substances in the emissions and extracts of each product category and then discuss how these data translate to the risk potential of products. Lastly, I discuss the scientific knowledge that can be derived from the available data and which questions have yet to be answered.

## Overview of SFPs

### E-cigarettes

An EC is a battery-powered device that heats a liquid, often containing nicotine, flavorings, and typically vegetable glycerin and propylene glycol to create an aerosol, which users then inhale. The first commercially successful device was created in 2003 by Hon Lik, a Chinese pharmacist, who aimed to create a product without combustion as a less harmful alternative to smoking. In detecting and quantifying the EC aerosol composition, one cannot deny the progress that has been achieved: recent studies report a substantial reduction (80–99.9%) in emission levels of HPHCs from modern ECs relative to cigarettes [19].

The nicotine used in EC-liquids is mostly extracted from tobacco, so the presence of trace levels of TSNAs detected by advanced trace analysis would not be surprising. However, recent analyses report a reduction of over 99.9% relative to reference cigarettes, where levels range from 185 to 3700 ng [19]. Among PAHs, combustible cigarettes deliver, on average, 6.7 ng of BaP [21],in contrast, levels of PAHs in EC emissions are not significant, especially when compared to ambient air (Threshold of PAH emissions in Germany: 1 ng/m^3^) with typical reductions ranging from 95.7 to 99.9% relative to combustible cigarettes. As a result, the toxicological risk posed by PAHs in EC is expected to be negligible.

### Heated tobacco products

Also known as heat-not-burn products, HTPs are electronic devices designed to heat tobacco while avoiding its combustion to produce a nicotine-containing aerosol with fewer and lower levels of HPHCs relative to cigarette smoke. The concept of heated tobacco has been around since the 1980 s, when initial prototypes heated the tobacco without burning using a piece of charcoal in an alumina tube. These prototypes were tested in my lab, where I had the privilege of analyzing the formation of PAHs under typical puffing conditions with a photoelectric aerosol sensor [18], of note, no PAHs were found in HTP emissions in those early unpublished studies.

Modern HTPs show a reduction in HPHC emissions of more than 93%, on average, as compared with a standard 1R6F laboratory cigarette [12]. Levels of the four TSNAs (NAB, NAT, NNK, and NNN) are reduced by 89–96%, while those of PAHs are reduced by 90%. As mentioned above, most of these compounds arise from the tobacco curing process, indicating that any remaining trace amounts of TSNAs or PAHs in HTP emissions may result from the tobacco used. Nevertheless, the toxicological risks from these compounds are likely to be substantially reduced as compared with cigarettes.

### Nicotine pouches

Similar to Swedish snus, NPs are small pouches that contain nicotine along with flavorings, sweeteners, and plant-based fibers. The pouch is neither heated nor burned, but placed between the gum and upper lip, where the nicotine is absorbed through the oral mucosa. NPs represent a further development of portion snus, tobacco-containing pouches traditionally used in Sweden and Norway that have established modified risk status [8].

An analysis of 46 NPs found that TSNAs were below the detection or quantification limit in extracts from most products [17]. The highest detected TSNA in one NP was NNN at 13 ng, which represents 0.35–10% of the amount detected in cigarette smoke. As would be expected for an oral nicotine product lacking tobacco, PAHs have not been found in NPs; furthermore, HPHC levels are similar to those in nicotine replacement therapies (NRTs) [4]. A recent evaluation of 20 NPs by the US Food and Drug Administration (FDA) confirmed that these SFPs have a significant reduction in HPHCs as compared with snus [9].

## Significance of reduced toxicant levels for relative harm potential

In Germany, the Federal Institute for Risk Assessment (BfR) plays a crucial role in consumer health protection, focusing on assessment and communication of risk. The BfR has conducted studies for all three of the SFPs discussed herein [15–17].

For ECs, the BfR has demonstrated that EC aerosols generally contain lower levels of HPHCs (e.g., formaldehyde, acetaldehyde, acrolein), while delivering nicotine levels comparable to those in cigarette smoke [16]. But ECs are not without risk: the aerosol from an EC clearly differs from that of a combustible cigarette. Nevertheless, well-established in vitro tests to determine the genotoxicity and mutagenicity of emissions reported negative results for EC aerosols but positive results for cigarette smoke; furthermore, the cytotoxicity of the EC aerosol is significantly reduced [7]. It is important to note, because it is often overlooked, that preclinical cell cultures exhibit responses to all constituents present in the aerosol or extracts. This comprehensive approach ensures that the evaluation encompasses the full spectrum of potential interactions and effects, thereby providing a more holistic understanding of the toxicological impact.

Similar results have been found for HTPs by the BfR, as well as by Dutch (RIVM) and American (FDA) public health bodies [22]. The BfR findings show that HTPs emit fewer and low levels of HPHCs relative to combustible cigarettes [15]; in particular, there are significant reductions in carcinogens like aldehydes and volatile organic compounds. These findings are consistent with in vitro toxicological studies, which support the reduced toxicity of HTP aerosol in comparison with cigarette smoke [6].

Lastly, as mentioned above, the BfR’s analysis of 46 NPs found that TSNAs were below the detection quantification limit in extracts from most products [17], while another study reported TSNA levels in the range of those within NRTs [4]. In vitro toxicological studies also indicate that NPs exhibit significantly lower mutagenicity, genotoxicity, and cytotoxicity compared to combustible cigarettes (e.g., [14]), while also providing sufficient amounts of nicotine, thereby appearing to be a potentially less harmful alternative for current smokers.

Interestingly, the BfR has published a statement confirming that NPs can reduce health risks relative to smoking. However, as the German state authorities currently classify NPs as a novel food which must be officially authorized, NPs are not marketable in Germany and are therefore unregulated. Nevertheless, they are easily available in the country. To ensure consumer protection, the BfR statement recommends the regulation of NPs, for example, with a nicotine limit of 16.7 mg per pouch.

Given the absence of long-term studies, the relative harm potential of SFP aerosols/extracts in vivo is being evaluated by biomarker studies. Biomarkers are measurable indicators that show whether certain toxicants have entered the body, whether biological processes are running normally, and/or whether there are signs of disease. Studies distinguish between biomarkers of exposure (BoE), which are associated with tobacco use (i.e., nicotine and cotinine) and biomarkers of potential harm (BoPH), which show the presence of harmful substances or altered metabolic pathways and thus provide an indication of potential health risks. In numerous studies of individuals using HTPs, ECs and NPs, exclusively or concurrently with combustible cigarettes (Dual Use) tobacco-associated biomarkers have been detected; however, the quantities are significantly reduced as compared with cigarette smoking, (e.g., [2, 3, 13]), supporting the in vitro evidence that SFPs are less harmful.

In addition, a mathematical model has estimated the theoretical cancer potencies of various SFPs based on their relative reductions in toxicant emissions compared to conventional cigarettes. According to this, the cancer potency of HTPs is approximately 40- to 50-fold lower than that of combustible cigarettes, while that of ECs is estimated to be 100- to 500-fold lower [23]. Given the substantially reduced chemical complexity of NPs, as reported by the BfR, it is reasonable to infer that the cancer potency associated with NPs is markedly reduced as well.

Overall, it seems highly likely to me that the reduction in toxicity observed for SFPs must translate—to some extent—to a reduction in risk for a smoker who switches.

## Research gaps and recommendations

As demonstrated above, SFPs have a significantly reduced toxicant profile as compared with combustible cigarettes. While they are not without risk and should be avoided by non-smokers, particularly the underage, SFPs provide a viable alternative for adult smokers who would otherwise continue smoking. Moreover, some SFPs exhibit levels of toxicants, including TSNAs and PAHs, that are comparable to or even lower than those found in endorsed NRTs, further underscoring their potential as harm reduction tools.

From my perspective, there is no doubt that SFPs can assist individuals to transition away from cigarette smoking in a more gradual manner. Use of SFPs would allow them to access nicotine without the health risks typically associated with traditional smoking. That said, it is important to identify key issues that will lead to further improvements regarding both the scientific assessment of SFPs and their reduced risk profile.

### Study design

There is an urgent need for longitudinal studies across all SFP categories to comprehensively assess their long-term effects on health and to support evidence-based regulatory decisions. These studies should also investigate the full spectrum of chemical constituents in aerosols, including unknown compounds, using non-targeted analysis techniques.

### Standardization of testing methods

Currently, there are very few standardized protocols for generating and analyzing HTP/EC-aerosol and NP samples. The International Organization for Standardization and the Cooperation Centre for Scientific Research Relative to Tobacco have developed specific standards for ECs and HTPs; however, as yet there are no established standards for testing NPs.

### Quality control

To further mitigate risks, quality control measures should be enhanced across all product categories. There is a significant demand for standardized and rigorous chemo-analytical quality control of all HTP components that meet heated surfaces. Tobacco, as the source of nicotine, flavor, and aroma in the sublimation process within an HTP, will exhibit varying strengths depending on the naturally occurring catalytic compounds present in the heated biomass.

For NPs and ECs, TSNAs can and should be eliminated, as suggested by the findings of the BfR and corroborated by Back et al. [4], who demonstrated the feasibility of producing highly pure nicotine products. Additionally, quality standards should establish nicotine concentration limits, ensuring product safety and consistency.

Further quality control of SFPs must integrate considerations of device and product characteristics, including their susceptibility to manipulation. In ECs, for example, variations in device temperature settings and liquid compositions can significantly influence the release of harmful substances. HTPs and NPs are less prone to such modifications, providing more consistent exposure profiles.

### Inclusion of industry in the scientific discourse

The involvement of the tobacco and nicotine industry in the scientific evaluation of SFPs warrants a balanced and critical approach. While concerns about conflicts of interest are valid, parallels can be drawn from the pharmaceutical sector, where industry-sponsored research is subject to established norms of transparency, peer review, and methodological scrutiny. Provided that similar standards are rigorously applied, contributions from the tobacco and nicotine industry may offer valuable data and insights. Encouraging participation in reputable scientific forums—such as those focused on aerosol science, toxicology, and analytical chemistry—could enhance interdisciplinary dialogue and promote a more comprehensive understanding of the evidence base surrounding SFPs.

## Summary and conclusion

There now exists a body of evidence regarding the toxicant profile and toxicological effects of SFPs. It is clear that ECs, HTPs, and NPs deliver nicotine with a reduced risk profile, making them a feasible alternative to combustible cigarettes. Although there seems to be a consensus about the risk continuum of various tobacco and nicotine products, where cigarettes are considered the most harmful of products, various countries draw different conclusions from this evidence. While the US authorizes certain heated tobacco products, e-cigarettes and nicotine pouches, the UK embraces a pragmatic harm reduction approach, German medical associations focus on an abstinence and prevention approach, emphasizing the potential harm from SFPs.

SFPs are not harmless: they contain nicotine, which has an addictive potential. However, SFPs emit only a fraction of the toxicants emitted by a combustible cigarette. Moreover, they can help people who smoke to transition towards using a less harmful nicotine product. Of course, protecting non-smokers and underaged individuals from nicotine use is fundamental, but this must not come at the expense of depriving adult smokers of access to safer alternatives like SFPs.

It is the nature of science that the questions will never end, and thus the evidence will never be complete; nevertheless, the totality of information and data should be considered when discussing the regulation and marketing of these tobacco and nicotine products. At present, this evidence points to SFPs playing a useful role in curbing the individual and societal risks associated with smoking.
